# The Effects of Heart and Skeletal Muscle Inflammation and Cardiomyopathy Syndrome on Creatine Kinase and Lactate Dehydrogenase Levels in Atlantic Salmon (*Salmo salar* L.)

**DOI:** 10.1100/2012/741302

**Published:** 2012-05-22

**Authors:** Muhammad Naveed Yousaf, Mark D. Powell

**Affiliations:** Faculty of Biosciences and Aquaculture, University of Nordland, 8049 Bodo, Norway

## Abstract

Heart and skeletal muscle inflammation (HSMI) and cardiomyopathy syndrome (CMS) are putative viral cardiac diseases of Atlantic salmon. This study examined the levels and correlated the serum enzymes creatine kinase (CK) and lactate dehydrogenase (LDH) to the histopathology of clinical outbreaks of HSMI and chronic CMS in farmed Atlantic salmon. A total of 75 fish from 3 different HSMI outbreaks, 30 chronic CMS fish, and 68 fish from 3 nondiseased fish groups were used as the study population (*N* = 173). Serum CK and LDH levels correlated significantly with the total inflammation and total necrosis scores for HSMI fish (*P* = 0.001). However, no correlation was identified for enzyme levels and histopathology scores for chronic CMS fish. The significantly increased CK and LDH levels and their positive correlations to histopathology differentiate HSMI from CMS clinically suggesting the potential use of enzymes for screening for HSMI is promising.

## 1. Introduction

The marine farmed Atlantic salmon (*Salmo salar *L.) exhibits a variety of cardiac diseases, and the reason for this likely includes low activity in relatively confined spaces, continuous food supply, low oxygen level, crowding, stress in handling, and temperature [1]. The cardiac anomalies and defects of Atlantic salmon include aplasia or hypoplasia of the septum transversum, abnormal location and shape of heart [1], arteriosclerosis [2, 3], and ventricular hypoplasia [4], but specific diseases include cardiomyopathy syndrome (CMS) [5–7], pancreas disease (PD) [8–10], and heart and skeletal muscle inflammation (HSMI) [11, 12]. Annual economical losses due to cardiomyopathy syndrome (CMS) alone have been estimated up to € 4.5–8.8 millions [7].

Heart and skeletal muscle inflammation (HSMI) is a disease of marine farmed Atlantic salmon reported from Norway, Scotland and Chile. HSMI is a disease which mainly affects heart and red skeletal muscle. It is typically a disease of moderate mortality (~20%) but high morbidity (~100%) that affects fish 5 to 9 months after transfer to sea. Presently, HSMI can be diagnosed by histopathology and presents as epi- and endocarditis as well as mononuclear cellular infiltration of both trabecular and compact layers of ventricle myocardium accompanied by myocytic necrosis [11–14]. HSMI is transmissible in laboratory studies by injecting tissue homogenate from diseased fish to healthy fish [11, 15], and recently piscine reovirus (PRV) has been suggested to be associated with HSMI infection [16, 17]. Lesions first appear and are more frequent in heart than red skeletal muscle. Affected myocytes show signs of degeneration, loss of cardiomyocytes striation and eosinophilia, loss of skeletal muscle striation, vacuolation, centralized nuclei, and karyorrhexis. There are more inflammatory changes as compared to necrotic changes in heart and red skeletal muscle [1, 12, 13]. HSMI has become more significant where outbreaks have increased from 54 in 2004 [18] to 162 cases reported in 2011 [19].

Cardiomyopathy syndrome (CMS) is a cardiac disease of Atlantic salmon with a suggested totiviral etiology [20] that mainly affects atrium and trabecular ventricle myocardium without involvement of skeletal muscle. It shares similar features with HSMI where both cause myocarditis [1]. Histopathological changes include necrosis and inflammation of trabecular layer of ventricle and atrium, epicarditis, cellular infiltrates of mainly mononuclear lymphocytes and macrophages, and rupture of atrium or sinus venosus macroscopically [1, 5]. CMS affects adult salmon after 12–18 months of sea transfer, and recently a totivirus (piscine myocarditis virus (PMCV)) is proposed as causative agent for cardiomyopathy [5, 17, 20, 21]. The piscine myocarditis virus is a double-stranded RNA virus with diameter of 50 nm and 6688 bp genome size [21]. The haematological tests and serum analysis for fish, compared with other areas of veterinary medicine, are not common place compared to higher vertebrates due to the lack of reference values for clinical chemistry (physiological and pathological) and understanding of disease pathogenesis and pathophysiology [22–24]. There is a strong need for blood biochemistry ranges especially where the etiology is unclear (HSMI and CMS) since the associated viruses can be present asymptomatically [17, 25]. Biochemical enzymes such as creatine kinase (CK) and lactate dehydrogenase (LDH) are well-established biomarkers of cardiac disease in humans [26] and are often used in conjunction with other hormonal biomarkers for a myocardial dysfunction diagnosis [27]. Both enzymes are released upon cellular degeneration such as necrosis. Lactate dehydrogenase (LDH) is involved in the interconversion of pyruvate and L lactate during the final reactions of glycolysis and is present in the cytoplasm of all cells (nucleated and nonnucleated cells). In humans, raised LDH plasma values are observed from 8 to 12 h, peaking within 2–3 days, and levels are sustained for duration of 7–10 days following cardiac injury [28]. Creatine kinase (CK), on the other hand, is found in the myocyte cytoplasm, sarcoplasmic reticulum, mitochondria, and myofibrils with a half-life of about 12 h in humans. Creatine kinase levels in blood plasma rise from 4 to 6 h peaking at 12–36 h and sustained over 3-4 days in humans where a cardiac injury has occurred [28]. The creatine kinase concentrations are related to the irreversible injury associated with myocardial necrosis in mammals (dogs) [29]. The elevated CK levels have been reported in association with myocytic necrosis seen in pancreas disease (PD) in Atlantic salmon [30, 31], and these results suggested that CK could be a useful candidate indicator of cardiac diseases in Atlantic salmon.

The aims of the present study were to measure the serum CK and LDH levels and examining their relationship to the cardiac diseases (HSMI and CMS) of Atlantic salmon.

## 2. Material and Methods

### 2.1. Fish Sampling

Seven groups of Atlantic salmon (*Salmo salar* L.) were involved in this study. All samples from diseased fish were taken from the farms diagnosed with both diseases by National Veterinary Institute, Oslo, Norway (NVI) and further histopathology was performed to confirm the disease diagnosis during the study. Group 1 (*n* = 28) consisted of fish (S1) collected from a confirmed HSMI outbreak at a sea site during the peak mortality of the HSMI episode. Group 2 (*n* = 16) comprised fish (S0) collected from a confirmed HSMI sea cage outbreak two months after peak mortality period. Group 3 (*n* = 31) included fish (S0) from a sea cage site collected during the early onset of a HSMI outbreak. The clinical phase of the disease has been defined as the time period with increased mortality at farm due to HSMI [32]. The disease phase was determined from the peak mortality time at the farm [16]. All diseased groups were in the sea phase of salmon production and opportunistic samples collected from diseased cages on each farm. All three diseased farms were widely distant from each other in Nordland county, Norway. Group 4 (*n* = 30) included chronic CMS infected fish (S2) and had confirmed CMS outbreak in the past, and sampling was performed 6 months after CMS outbreak. Diseased groups included fish with average weight range (600–1000 g) for HSMI and (6000–7000 g) for CMS fish.

Group 5 (*n* = 28) was nondiseased fish which were taken from a study where Atlantic salmon (S0) had been made anaemic using phenylhydrazine, and cardiac hypertrophy had been characterized [33]. Group 6 (*n* = 20) consisted of nondiseased, apparently healthy fish (S0 + S1) kept in a laboratory facility (University of Nordland, Mørkvedbukta Research Station, Bodo, Norway) in 2 m³ tanks with fresh ambient sea water (temperature range 7-8°C) and fed 0.7% commercial feed (Spirit, Skretting, Stavanger, Norway) of their body weight three times weekly. Group 7 (*n* = 20) comprised of apparently healthy, nondiseased Atlantic salmon (S1) from the sea cages. Nondiseased groups included fish with average weight range (400–2000 g).

### 2.2. Blood Collection

All fish were killed by a blow to the head or overdose of tricaine methanesulfonate (MS222) (100 mg mL^−1^). Blood was collected immediately from the caudal vein with a 5 mL syringe using 23 G needle, allowed to clot in Eppendorf tubes for 2–4 h, centrifuged at 8,000 g for 5 min, and the serum collected except group 4 where heparinised blood plasma was collected and frozen at −20°C.

### 2.3. Serum Analysis

All samples were frozen and sent on dry ice to Norwegian School of Veterinary Sciences, Oslo Central Laboratory and to the Nordland Hospital, Department of Medicine Biochemistry, Bodo for creatine kinase (CK) and lactate dehydrogenase (LDH) analysis. Creatine kinase (CK) and lactate dehydrogenase (LDH) were measured by using ADIVA 1650 (Siemens Medical Solution Diagnostics Inc., Tarrytown, NY, USA) at Norwegian school of veterinary sciences, Oslo central laboratory and ADVIA 1650/1800 (Bayer Diagnostics, Tarrytown, NY, USA) at Nordland Hospital, Department of Medicine Biochemistry, Bodo on the basis of their enzyme activity and measured by increase in absorbance at 340/410 nm. Both laboratories used the same methods. Briefly the principle of the procedures for LDH and CK is as follows: LD catalyzes the conversion of L lactate to pyruvate in the presence of nicotinamide adenine dinucleotide (NAD). The enzyme activity of LD is proportional to the rate of production of NADH (reduced NAD). Creatine kinase reacts with creatine phosphate and ADP to form ATP which is coupled to the hexokinase-G6PD reaction, generating NADPH. The concentrations of NADH and NADPH were measured by the increase in absorbance at 340/410 nm for LDH and CK, respectively.

### 2.4. Histopathology and Scoring Method

To correlate biochemical enzymes (CK and LDH), histopathology was method of choice for diagnosis of HSMI. Hearts, skeletal red and white muscle from below the dorsal fin and above the lateral line, and other vital organs, were collected and fixed in 10% neutral phosphate-buffered formalin solution. External and internal visual examination was performed in addition to histological observation of other vital organs for other abnormalities or signs of overt disease in the fish. Tissues were processed by a standard paraffin wax protocol (dehydrated, embedded in paraffin, 3 *μ*m thick sectioned and H and E stained) and examined for changes (necrosis and inflammation) characteristic of HSMI [11]. The case definition for HSMI includes inflammation and necrosis of trabecular and compact ventricle myocardium, epicarditis, endocarditis, mononuclear inflammatory cell infiltration, and a higher level of inflammation compared with necrosis while supportive signs may also include inflammation and necrosis of red skeletal muscle, atrium, and absence of pancreatic lesions [32]. A semiquantitative assessment of each slide was adapted from McLoughlin et al. [34] for scoring histopathological findings in heart and muscle tissues obtained from seven groups of fishes used in this study. It has been used and established in PD, a similar cardiac disease to HSMI and CMS (Table 1).

### 2.5. Different Anatomical Regions

The scoring method was used for the detailed study of the tissue in anatomically distinct areas of the heart and skeletal muscle: (1) atrial trabecular myocardial inflammation, (2) and necrosis, (3) atrial epicarditis, (4) ventricle compact myocardial inflammation, (5) and necrosis, (6) ventricle trabecular myocardial inflammation, (7) and necrosis, (8) ventricle epicarditis, (9) skeletal muscle inflammation, (10) and necrosis. A total inflammation score was determined from the summed scores of all parameters (atrial and ventricular trabecular inflammation, atrial and ventricular epicarditis, ventricle compact inflammation, and skeletal muscle inflammation) and total necrosis score determined from the summed scores of all parameters (atrial and ventricular trabecular necrosis, ventricle compact necrosis, and skeletal muscle necrosis). The sum scores of inflammation and necrosis in heart and skeletal muscle were correlated with the biochemical enzymes (CK and LDH) levels. Total inflammation and total necrosis scores were correlated to the HSMI plus nondiseased fish and the CMS fish plus nondiseased fish to differentiate the enzymatic effects in each disease separately.

### 2.6. Slides Evaluation

Slides were evaluated blindly by two persons, histopathological results compared between the groups and correlated with respective biochemical enzymes values for each sample. Sometimes the conflict for slide score was around 0.5–1 between two persons, and then they agreed after discussing case definition and scoring system on most suitable score for the slide.

### 2.7. Statistical Analysis

Spearman rank coefficient correlations were performed using SigmaPlot (10.0) and were considered statistically significant at *P* values ≤0.05. The mean histopathology (total inflammation and total necrosis scores) and enzyme (CK and LDH) values were analyzed using Kruskal-Wallis One Way Analysis of Variance on ranks with differences isolated using Dunn's post hoc analysis. Data were presented as mean ± SD.

## 3. Results

The highest and lowest mean CK values were identified in HSMI fish group 1 =  16479.25 ± 1844.49 IU.L^−1^ and nondiseased fish group 5 =  1581.71 ± 425.33 IU.L^−1^ respectively. However, the highest and lowest mean LDH values were identified in HSMI fish group 2 =  1838.25 ± 957.47 IU.L^−1^ and nondiseased fish group 5 =  235.39 ± 27.43 IU.L^−1^, respectively. The mean CK and LDH values for CMS fish were (5207.93 ± 967.81 IU.L^−1^) and (426.2 ± 60.68 IU.L^−1^), respectively (Table 4).

Diseased group 1 (HSMI fish) had significantly the highest levels for CK activity as compared to other HSMI groups 2 and 3 (Kruskal-Wallis One-Way Analysis: H = 65.217, d.f. = 6; (*P* ≤ 0.001) (Figure 2(a)). However, LDH activity levels were higher in HSMI fish (group 3) as compared to other HSMI fish (groups 1 and 2) (Kruskal-Wallis One-Way Analysis: H = 73.838, d.f. = 6;  *P* ≤ 0.001) (Figure 2(b)). The CMS (group 4) CK and LDH values were significantly different from nondiseased fish (groups 5, 6, and 7) (Figures 2(a) and 2(b)).

**Figure 1:**
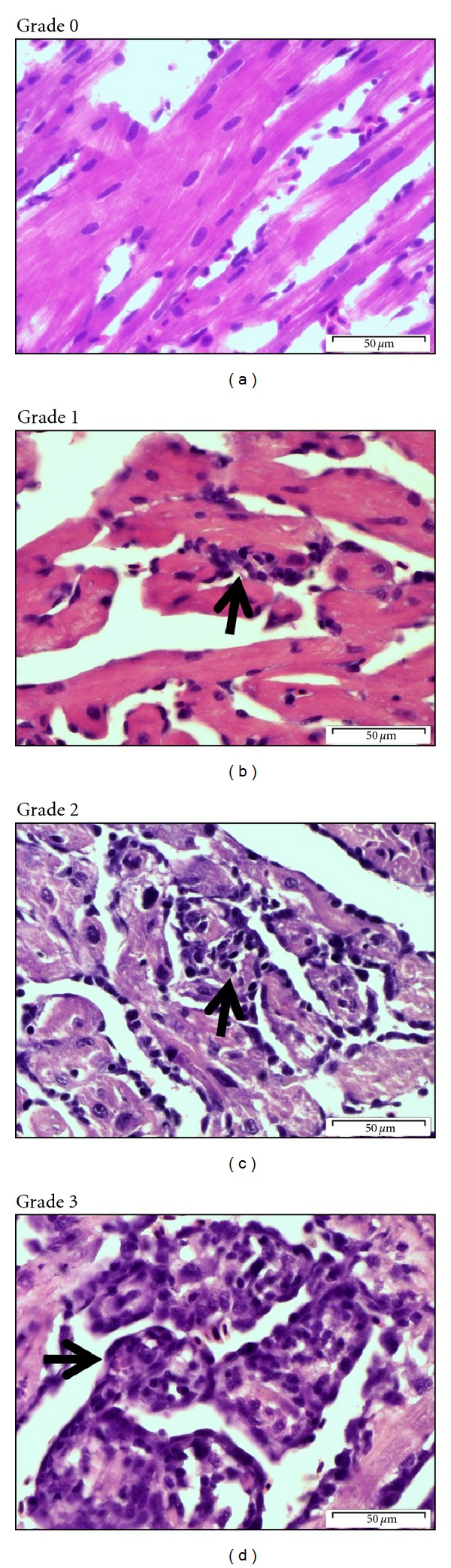
The representative micrographs of the semiquantitative scoring system described in Table 1, represented the heart histopathology. Grade 0: no pathological changes. Grade 1: minor inflammatory lesions comprises of focal subendocardial mononuclear leukocytes. Grade 2: several distinct lesions with moderately increased number of mononuclear leukocytes. Grade 3: severe lesions where almost all myofibres have been replaced by inflammatory cells, predominantly by mononuclear lymphocyte-like cells. Arrow: inflammation. Scale bars = 50 *μ*m.

**Figure 2:**
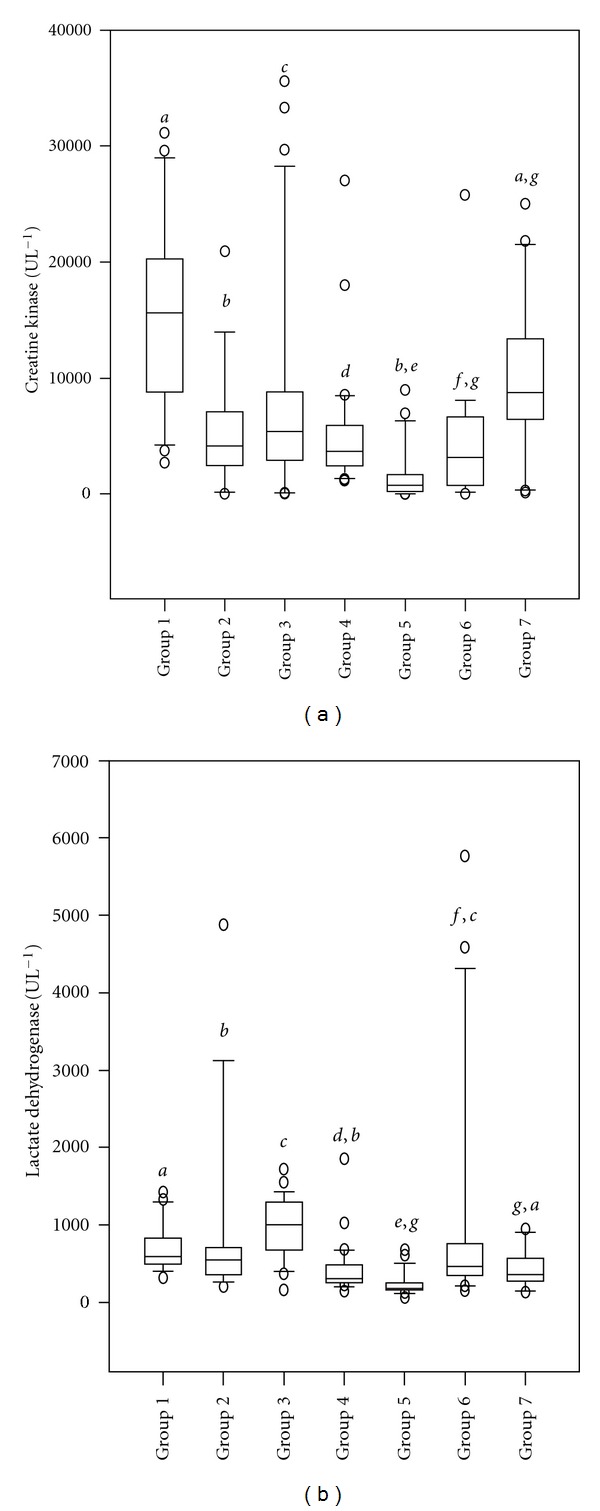
The box plots of (a) creatine kinase (CK) and (b) lactate dehydrogenase (LDH) enzymes activity values in the serum/plasma of Atlantic salmon for all seven groups (*N* = 173). Group 1 (*n* = 28) and group 2 (*n* = 16) represented fish from an acute and late phase of a HSMI outbreak, respectively, while group 3 (*n* = 31) represented values from fish from an early phase of HSMI. Group 4 (*n* = 30) included chronic CMS fish. Group 5 (*n* = 28), group 6 (*n* = 20), and group 7 (*n* = 20) represented values from nondiseased fish. Bars with different letters represented significant differences between groups (*P* < 0.05). (∘) denotes outliers.

The scoring grades which were used to score the inflammatory changes represented by micrographs (Figure 1) and reflected the semiquantitative scoring system which was applied to each anatomical region of the heart (atrium, compact and trabecular ventricular myocardium, pericardium) and the red skeletal muscle (Table 1).

**Figure 3:**
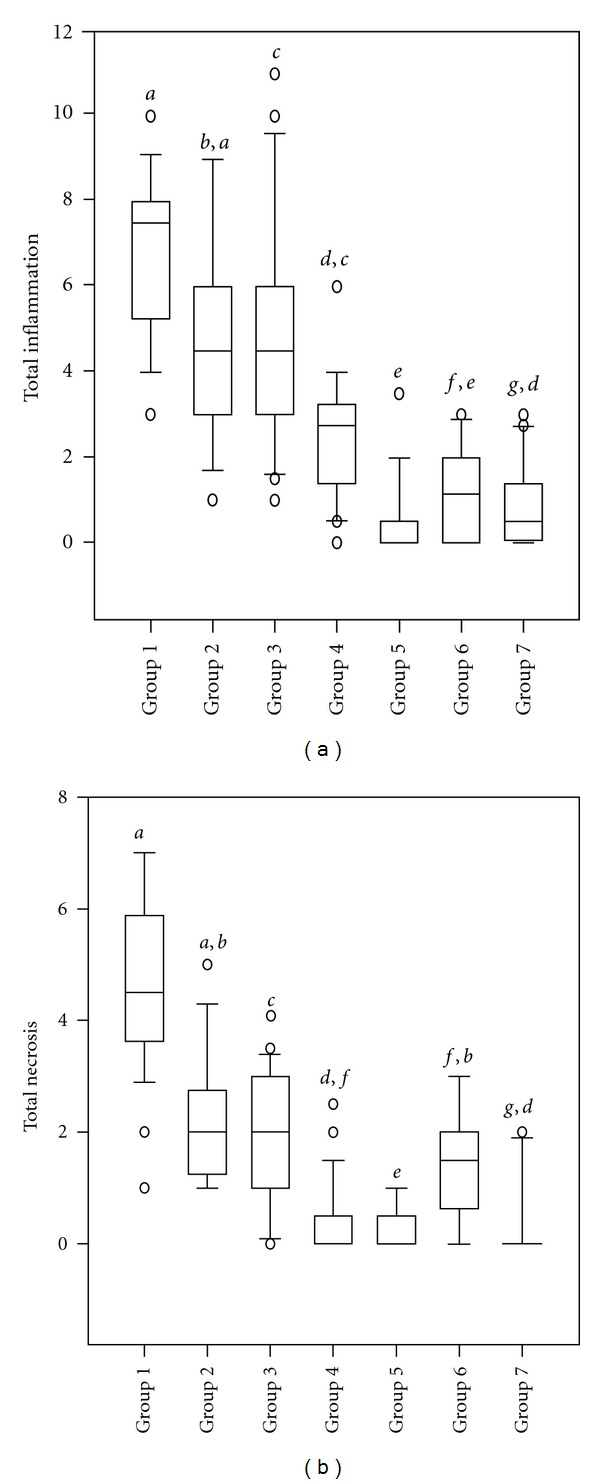
The box plots of all seven groups of Atlantic salmon on the basis of (a) total inflammation and (b) total necrosis in the heart and skeletal muscle (*N* = 173). Group 1 (*n* = 28) and group 2 (*n* = 16) represented fish from an acute and late phase of a HSMI outbreak, respectively, while group 3 (*n* = 31) represented values from fish from an early phase of HSMI, and group 4 (*n* = 30) included chronic CMS fish. Group 5 (*n* = 28), group 6 (*n* = 20), and group 7 (*n* = 20) represented values from nondiseased (non-HSMI) fish. Higher scores of inflammation and necrosis were found in diseased (groups 1, 2, 3, and 4) fish while low levels of scores in nondiseased (groups 5, 6, and 7) fish were present. Bars with different letters represented significant differences between groups (*P* < 0.05). (∘) denotes outliers.

Seven fish groups were compared on the basis of inflammation and necrosis scores. Inflammatory mononuclear cells were more frequent as compared to focal areas of necrosis in heart tissue, but the opposite was apparent in the red skeletal muscle where necrosis predominated. The mean total inflammation (Kruskal-Wallis One-Way Analysis: H = 111.216, d.f. = 2; (*P* ≤ 0.001)  and total necrosis (Kruskal-Wallis One-Way Analysis: H = 90.484, d.f. = 2; (*P* ≤ 0.001)  scores of HSMI and CMS fish were significantly different from nondiseased fish with the exception of total necrosis scores of CMS fish which were not significantly different from nondiseased fish (Table 2). More lesions were present in the heart as compared to the skeletal muscle (Table 2). Total inflammation scores ranged from 1 to 11 in diseased groups while 0 to 3.5 in nondiseased groups. Total necrosis scores ranged from 0 to 7 for diseased groups while from 0 to 3 for nondiseased groups. Total necrosis (Kruskal-Wallis One-Way Analysis: H = 118.135, d.f. = 6; (*P* ≤ 0.001) (Figure 3(b)) and total inflammation (Kruskal-Wallis One-Way Analysis: H = 119.558, d.f. = 6; (*P* ≤ 0.001) (Figure 3(a)) scores were compared for all seven groups and identified higher scores in HSMI group 1 while being significant and lower in the group 2 and 3 as compared to none to low level of changes seen in nondiseased fish (groups 5, 6, and 7) (Figures 3(a) and 3(b)). In general, muscle necrosis and inflammation (cardiac and skeletal) were negligible to mild in nondiseased fish (groups 5, 6, and 7) as compared to CMS and HSMI fish (groups 1, 2, 3, and 4). Total inflammation and total necrosis results of all HSMI-diseased fish (groups 1, 2, and 3) were significantly different from nondiseased groups (groups 5, 6, and 7) with the exceptions of total necrosis scores of CMS fish (group 4) which was not significantly different from nondiseased fish (group 6) (Figures 3(a) and 3(b)).

The histopathology scores (sum score of heart and muscle necrosis and inflammation) were correlated with serum/plasma CK and LDH levels. The correlations which were made among CK enzyme levels, and different anatomical parameters of all fish groups excluding CMS fish (group 4) gave significant relationships (*P* ≤ 0.001) (Table 3). The significant correlations of CK levels to individual parameters included atrial inflammation (S coeff. = 0.451, *P* < 0.001), atrial necrosis (S coeff. = 0.252, *P* = 0.002), atrial epicarditis (S coeff. = 0.314, *P* < 0.001), ventricle compact layer inflammation (S coeff. = 0.440, *P* < 0.001), ventricle compact layer necrosis (S coeff. = 0.249, *P* = 0.002), ventricle trabecular inflammation (S coeff. = 0.526, *P* < 0.001), ventricle trabecular necrosis (S coeff. = 0.283, *P* < 0.001), ventricle epicarditis (S coeff. = 0.333, *P* < 0.001), skeletal muscle necrosis (S coeff. = 0.206, *P* = 0.035), and skeletal muscle inflammation (S coeff. = 0.169, *P* = 0.084). The CK enzyme levels significantly and positively correlated with the both total inflammation (S coeff. = 0.552, *P* < 0.001) and total necrosis (S coeff. = 0.526, *P* < 0.001) scores (Table 3). The LDH levels were also correlated in the same manner as above for CK which showed significant relationships (*P* = 0.05) (Table 3). The significant correlations for LDH levels to different parameters were atrial inflammation (S coeff. = 0.254, *P* = 0.002), ventricle compact layer inflammation (S coeff. = 0.297, *P* = 0.001), ventricle trabecular inflammation (S coeff. = 0.166, *P* = 0.049), skeletal muscle inflammation (S coeff. = 0.373, *P* = 0.001), ventricle epicarditis (S coeff. = 0.20, *P* = 0.016), and skeletal muscle necrosis (S coeff. = 0.414, *P* = 0.001) (Table 3). However, few non-significant relationships were identified for LDH and anatomical parameters such as ventricle compact layer necrosis (S coeff. = 0.080, *P* = 0.341), ventricle trabecular necrosis (S coeff. = 0.052, *P* = 0.534), atrial necrosis (S coeff. = 0.034, *P* = 0.688), and atrial epicarditis (S coeff. = −0.049, *P* = 0.558). There were significant positive correlations between LDH levels and the total inflammation (S coeff. = 0.266, *P* < 0.001) and total necrosis (S coeff. = 0.247, *P* < 0.003) scores (Table 3). The correlations between CK enzyme levels and different anatomical parameters of all fish groups excluding HSMI fish (groups 1, 2, and 3) identified non-significant relationships (*P* = 0.05) (Table 3). The correlations made between CK levels and different anatomical regions were atrial inflammation (S coeff. = 0.108, *P* = 0.580), atrial necrosis (S coeff. = 0.327, *P* = 0.077), ventricle trabecular inflammation (S coeff. = 0.157, *P* = 0.405), ventricle trabecular necrosis (S coeff. = 0.198, *P* = 0.291), ventricle epicarditis (S coeff. = 0.340, *P* = 0.065), and skeletal muscle necrosis (S coeff. = 0.068, *P* = 0.719). The LDH levels were also correlated in the same manner as above for CK and included atrial inflammation (S coeff. = 0.052, *P* = 0.790), atrial necrosis (S coeff. = 0.242, *P* = 0.195), ventricle trabecular inflammation (S coeff. = −0.043, *P* = 0.822), ventricle trabecular necrosis (S coeff. = 0.122, *P* = 0.516), ventricle epicarditis (S coeff. = 0.309, *P* = 0.096), and skeletal muscle necrosis (S coeff. = −0.024, *P* = 0.899). The combined CMS and nondiseased group's correlations with CK levels were also made in the same manner as described above and identified correlations for total inflammation (S coeff. = 0.089, *P* = 0.635) and total necrosis (S coeff. = 0.355, *P* = 0.075) scores (Table 3). There were non-significant negative and positive correlations between LDH levels and total inflammation and total necrosis for CMS group, respectively (Table 3).

## 4. Discussion

The CK and LDH values of all seven fish groups were compared and diseased fish (HSMI) identified with significantly higher enzymes levels as compared to nondiseased fish. The significantly higher and lower mean enzymes levels in diseased and nondiseased fish, respectively, were consistent with the CK enzyme ranges already reported in farmed Atlantic salmon affected with a similar pancreas disease (PD) [30, 31]. The highest mean LDH levels were identified at the earlier phase of HSMI disease while highest CK levels were present in acute phase of the disease. Previous *in vivo* studies identified the increased CK and LDH activities in Atlantic salmon and Nile tilapia (*Oreochromis niloticus*) treated with tributyltin (TBT) and cadmium, respectively [35, 36].

Histopathology was used as a method of choice to diagnose the diseases (HSMI and CMS). This study described the histopathology in the heart and skeletal muscle by using a semiquantitative scoring system that addressed the pathological changes in both tissues (cardiac and somatic muscle). The diseased fish showed the histopathological changes in the heart and skeletal muscle similar to HSMI and in hearts for CMS fish [5, 11, 13]. The histological changes were identified in both atrium and ventricle (compact and trabecular) of HSMI fish while mostly ventricular trabecular layer was involved in CMS fish. The histopathological changes were compared for all seven fish groups, and hearts were identified with most tissue damage and suggested to be the contributing source of enzymes (CK and LDH) which released upon cellular damage and in line with Rodger et al. [30] that suggested the significantly higher CK levels due to myopathy in PD-affected Atlantic salmon.

The mean CK levels and histopathology scores for acute phase HSMI fish (group 1) were doubled than early or late phase of HSMI fish (groups 2 and 3), and mean CK levels (group 1) were up to four times greater than nondiseased fish (groups 5 and 6). These higher CK levels and total inflammation scores were suggested to be the disease (HSMI) outcome and supported the notion that fish included in group 1 were in the acute phase of disease whereas groups 2 and 3 were not in clinical phase of a HSMI outbreak. The acute phase of the disease (HSMI) corresponded to higher mortality rates on the farm and creatine kinase levels in blood sustained over 3-4 days in humans where a cardiac injury has occurred [28].

The HSMI-infected fish showed significantly higher histopathological scores as compared to the nondiseased fish. The higher histopathological scores were consistent with the higher CK levels in diseased fish as compared to nondiseased fish, supported the higher enzymes levels likely due to myopathy. The total inflammation scores were doubled as compared to the total necrosis scores in all HSMI fish which were considered as clinical sign of HSMI while necrosis being suggested as a secondary effect [13, 15]. The mean CK and LDH levels and total necrosis scores for chronic CMS fish were equal or lower to the nondiseased fish suggested no correlation to CMS fish and supported the hypothesis that increased enzymes levels identified in the HSMI fish were related to myopathy [30, 31]. The fish cages that experienced the CMS outbreak showed high inflammation scores as compared to other cages on the same farm that were not diagnosed with CMS, and both CMS and non-CMS fish had higher values of enzymes indicating that CK and LDH were not correlated to the chronic CMS fish histopathology scores.

The significantly positive correlations were identified with biochemical enzymes (CK and LDH) and histopathological changes in HSMI-affected Atlantic salmon [12, 24]. The histopathological scores for anatomically distinct areas of heart and skeletal muscle were correlated significantly with respective CK and LDH levels of the fish (*P* = 0.05). The serum enzymes (CK and LDH) correlations have been used previously to find significant relationships in great sturgeon (*Huso huso*) and rainbow trout (*Oncorhynchus mykiss*) [24, 37, 38]. The correlations between histopathology (inflammation and necrosis) and enzymes (CK and LDH) values were significant and positive, and suggested the HSMI disease effects on blood biochemistry of Atlantic salmon and consistent with mammalian studies where blood biochemistry is changed in pathological conditions and used to predict the disease [29, 36]. The correlation of CK levels to HSMI histopathology appeared useful due to the release of CK after tissue injury and potential contribution in limited piscine blood biochemistry [28]. However, the CMS group showed non-significant correlations between histology and enzymes and suggested that blood biochemistry of Atlantic salmon might not be affected due to chronic CMS disease. Another reason for no correlation of chronic CMS fish might include the time of fish sampling which was conducted 6 months after acute phase of disease while biochemical enzymes (CK and LDH) release rapidly following tissue injury with peak levels for 10–12 days in human [28]. It was also supported by the fact that chronic CMS fish did not show signs (higher mortality levels) and severe histopathological lesions characteristic of acute phase of disease suggesting the late or chronic phase of disease. However, further studies are required to completely understand the CMS disease effects on the blood biochemistry. Histopathology is still a diagnostic method of choice in clinical case of HSMI even though the recent identification of a reovirus associated with the disease allows the possibility of identifying infected animals. However, asymptomatic Atlantic salmon have been identified with piscine reovirus and piscine myocarditis virus by RT-qPCR [17, 25]. However, it is a terminal procedure performed after the onset of disease and there has been as observed increase mortality level on farm, and mis-diagnosis may result with diseases such as PD and CMS presenting similar pathological changes to HSMI [12]. The blood biochemistry tests may prove useful and have been proved useful in the detection and diagnosis of metabolic disturbances in a number of diseases [39]. The use of serum enzymes to diagnose the cardiac diseases in humans is a common practice and a well-established method. The CK and LDH levels are affected in the cardiac diseases and serve as disease indicators in humans [26]. Previous attempts at measuring CK values were made in similar disease such as pancreas disease (PD) but were not correlated directly and extensively with histopathology [30, 31]. These studies have been shown the significantly increased CK levels and suggested the tissue damage as source of enzyme. HSMI fish exhibited higher mean histopathology scores and enzymes (CK and LDH) levels, and serum enzymes showed significant positive correlations to histopathology which supported the notion that CK and LDH levels were affected due to natural HSMI outbreak. The use of CK and LDH enzymes haematological levels for pathological changes at least for HSMI appeared promising and a potential contribution in the limited piscine blood biochemistry by identifying the enzyme ranges in the above-mentioned fish groups [22].

In conclusion, the present study measured the CK and LDH levels in diseased and nondiseased Atlantic salmon and correlated significantly to the histopathology of Atlantic salmon affected with natural HSMI outbreaks while being non-significant to chronic CMS. The significantly higher CK levels correlated positively and significantly to HSMI pathological changes, suggesting that the potential use of serum enzymes for screening HSMI is promising. The findings of the present study should be considered as a contribution to the more extensive research necessary to understand biological activities (enzymes) and the pathological changes of Atlantic salmon.

## Figures and Tables

**Table 1:** Semiquantitative lesion scoring system adapted from McLoughlin et al. [34]. System covers heart and skeletal muscle lesions separately. Lesions starting from 0 (healthy tissue) to 3 (severe changes). (a) Heart lesion classification. (b) Skeletal muscle classification.

Score	Description
(a)	
0	Normal appearance
1	Focal myocytic necrosis ± inflammation (<50 fibers affected)
2	Multifocal myocytic necrosis ± inflammation (50–100 fibers affected)
3	Severe diffuse myocytic necrosis ± inflammation (>100 fibers affected)

(b)	
0	Normal appearance
1	Focal myocytic necrosis ± inflammation
2	Multifocal myocytic necrosis ± inflammation
3	Severe diffuse myocytic necrosis ± inflammation

**Table 2:** Total mean (±  SD) scores of inflammation and necrosis for HSMI (groups 1, 2, and 3), CMS (group 4), and nondiseased fish (groups 5, 6, and 7) in heart and heart with skeletal muscle. Different letters represent significant differences between groups (*P* < 0.05).

Parameter	Nondiseased	HSMI	CMS
*Inflammation*			
Heart	0.83 ± 0.30	5.30 ± 1.16	2.44 ± 1.39
Heart + muscle	0.99 ± 0.16^a^	5.59 ± 1.03^b^	2.44 ± 1.39^c^

*Necrosis*			
Heart	0.35 ± 0.36	2.01 ± 1.34	0.1 ± 0.40
Heart + muscle	0.62 ± 0.45^a, c^	2.92 ± 1.15^b^	0.38 ± 0.68^c^

**Table 3:** The Spearman correlation coefficient for anatomically distinct regions of the HSMI and CMS infected fish to creatine kinase (CK) and lactate dehydrogenase (LDH). *P* values given in parentheses.

Parameter	HSMI		CMS	
	CK	LDH	CK	LDH
Ventricle compact necrosis	0.249 (0.002)	0.080 (0.341)	—	—
Ventricle trabecular necrosis	0.283 (<0.001)	0.052 (0.534)	0.198 (0.291)	0.122 (0.516)
Atrium necrosis	0.252 (0.002)	0.034 (0.688)	0.327 (0.077)	0.242 (0.195)
Skeletal muscle necrosis	0.206 (0.035)	0.414 (0.001)	0.068 (0.719)	−0.024 (0.899)
Ventricle compact inflammation	0.440 (<0.001)	0.297 (0.001)	—	—
Ventricle trabecular inflammation	0.526 (<0.001)	0.166 (0.049)	0.157 (0.405)	−0.043 (0.822)
Atrial inflammation	0.451 (<0.001)	0.254 (0.002)	0.108 (0.580)	0.052 (0.790)
Ventricle epicarditis	0.333 (<0.001)	0.20 (0.016)	0.340 (0.065)	0.309 (0.096)
Atrium epicarditis	0.314 (<0.001)	−0.049 (0.558)	—	—
Muscle inflammation	0.169 (0.084)	0.373 (0.001)	—	—

Total inflammation	0.552 (<0.001)	0.266 (<0.001)	0.089 (0.635)	−0.075 (0.691)

Total necrosis	0.526 (<0.001)	0.247 (0.003)	0.355 (0.075)	0.240 (0.209)

**Table 4:** Blood serum enzymes in different fish groups (Mean ± SE).

	Creatine kinase (IU.L^−1^)	Lactate dehydrogenase (IU.L^−1^)
Group 1	16479.25 ± 1844.49	697.43 ± 56.61
Group 2	10280 ± 5246.18	1838.25 ± 957.47
Group 3	8333.34 ± 1709.08	966 ± 71.94
Group 4	5207.93 ± 967.81	426.2 ± 60.68
Group 5	1581.71 ± 425.33	235.39 ± 27.43
Group 6	7098.35 ± 2916.95	1027.9 ± 334.04
Group 7	10297.15 ± 1531.11	423.3 ± 53.06
